# Targeting m^6^A RNA Modification in Tumor Therapeutics

**DOI:** 10.3390/curroncol32030159

**Published:** 2025-03-11

**Authors:** Zhenwei Mao, Min Li, Shengjun Wang

**Affiliations:** 1 Department of Laboratory Medicine, Affiliated People’s Hospital, Jiangsu University, Zhenjiang 212002, China; 2 Jiangsu Key Laboratory of Laboratory Medicine, Department of Immunology, School of Medicine, Jiangsu University, Zhenjiang 212002, China; 3 Department of Laboratory Medicine, Affiliated Hospital of Jiangsu University, Zhenjiang 212001, China

**Keywords:** N6-methyladenosine (m^6^A), mRNA, tumor, targeting therapy

## Abstract

The prevalent eukaryotic RNA modification N6-methyladenosine (m^6^A), which is distributed in more than 50% of cases, has demonstrated significant implications in both normal development and disease progression, particularly in the context of cancer. This review aims to discuss the potential efficacy of targeting tumor cells through modulation of m^6^A RNA levels. Specifically, we discuss how the upregulation or downregulation of integral or specific targets is effective in treating different tumor types and patients. Additionally, we will cover the factors influencing the efficacy of m^6^A RNA targeting in tumor treatment. Our review will focus on the impact of targeting m^6^A mRNA on genes and cells and assess its potential as a therapeutic strategy for tumors. Despite the challenges involved, further research on m^6^A RNA in tumors and its integration with existing tumor therapy approaches is warranted.

## 1. Introduction

N6-methyladenosine (m^6^A), as the most prevalent internal modification, is catalyzed by the METTL3/METTL14/WTAP complex at the pre-RNA level before its release from chromatin, with METTL3 serving as the core enzyme, METTL14 stabilizing and positioning the complex, and WTAP assisting in proper localization and function [1,2]. m^6^A has been implicated as a pivotal regulator of mRNA biology in normal development or in tumorigenesis [3,4,5,6,7]. m^6^A is found in various nucleic acids, although the specific composition varies (see Table 1), potentially due to differences in the localization and structural compatibility between enzymes and sequences. The m^6^A mRNA methylation process is a dynamic and reversible modification that is regulated by three key factors. These factors include methyltransferases, known as “writers”, such as METTL3, METTL14, WTAP, RBM15, ZC3H13, and KIAA1429 (VIRMA). METTL3 serves as a core subunit with catalytic activity, while METTL14 plays a role in substrate recognition. WTAP is responsible for recruiting METTL3 and METTL14, as well as interacting with other components to form hybrids. One-third of mRNAs are m^6^A-modified by the METTL3-METTL14-WTAP complex, which regulates mRNA maturation and translation. Additionally, “erasers” such as FTO and ALKBH5, which share a similar core domain structure, are able to demethylate RNA. Methylated RNA is identified by various proteins for the purpose of executing distinct biological activities. These proteins, known as “readers”, encompass YTHDC1, YTHDC2, YTHDF1, YTHDF2, and HNRNPC, among others. Proteins containing the YTH domain exhibit a specific affinity for methylated mRNA, thereby governing subsequent processes of translation and degradation. In the context of potential tumor treatment strategies, a comprehensive understanding of RNA and cell fate through m^6^A modification is imperative. Currently, the precise effects of targeting m^6^A mRNA in tumors remain ambiguous, necessitating further clarification to enhance the efficacy of therapeutic approaches.

## 2. Influence of m^6^A on mRNA Fate

Research has demonstrated that only a subset of RRACH (R = G or A; H = A, C, or U) sites within adenosine residues are methylated by m^6^A [14], which might indicate the importance of the tertiary structure domain of mRNA and the selective binding sites within the m^6^A enzyme–mRNA complex in determining the m^6^A methylation levels [15]. The impact of m^6^A modification on the fate of m^6^A mRNA is influenced by various m^6^A readers, with the different readers binding to it being a significant factor. While a range of m^6^A readers have been shown to have important effects on mRNA fate, with some promoting mRNA degradation, such as YTHDF2 [16], others have been reported to have beneficial effects on mRNA translation, including mRNA structure switching (HNRNPC, HNRNPG) [17,18,19], mRNA splicing (YTHDC1) [20,21], nuclear export (YTHDC1), stability (IGFBP1/2/3, PRRC2A, FMRP) [22], and translation (YTHDF1/3, IGFBP1/2/3, YTHDC2, elF3) [23].

One critical factor influencing the selectivity of m^6^A readers is the presence of m^6^A sites, which play a significant role in determining the selection of readers and the fate of mRNA. For instance, the terminator-m^6^A site is more likely to be recognized by YTHDF1, leading to enhanced translation efficiency with elF3, whereas the CDS-m^6^A site tends to interact with YTHDC2 [24]. Additionally, the recently discovered reader eIF3, which enhances mRNA translation, has been observed to bind to a single 5′ UTR m^6^A site to independently initiate translation [25]. Additionally, research has indicated that YTHDF2 plays a role in the preservation of 5′ UTR-m^6^A mRNA translation by competitively interacting with FTO [26], rather than facilitating m^6^A mRNA degradation at the 3′ UTR [25]. Studies have shown that transcripts of genes with moderate expression levels are more likely to undergo m^6^A modification compared to genes expressed at extreme levels [13,14,27]. Thus, the localization of m^6^A on mRNA at the 3′ UTR site and the overall mRNA expression level in cells are significant factors influencing mRNA fate.

Furthermore, the involvement of m^6^A sites and the fate of m^6^A mRNA, as well as the expression of the m^6^A mRNA methylase METTL3, are becoming increasingly significant factors in mRNA translation, facilitated by the collaboration of the translation machinery. It is widely acknowledged that certain readers, specifically YTHDF1 [28] and YTHDF2 [29], have the capability to directly interact with translation initiation proteins, such as elf3, and potentially influence their expression in order to regulate m^6^A mRNA translation. Surprisingly, recent findings have indicated that METTL3 has the ability to localize at the transcriptional start sites of numerous actively transcribed genes, where it collaborates with CEBPZ, a CAATT-box binding protein, to facilitate translation by alleviating ribosome stalling [30]. Additionally, METTL3 was also found to enhance the translation of oncogenes EGFR and TAZ in cancer cells by directly recruiting the translation initiation factor elf3, independent of its catalytic activity or any m^6^A readers. The necessity of an m^6^A mRNA reader for METTL3-mediated regulation remains uncertain, and further research is required to determine whether this process is a normal part of cell development or a response to tumor stress.

## 3. Influence of m^6^A mRNA on Cell Fate

### 3.1. Influence of m^6^A mRNA on Normal Cell Fate

In a normal state, METTL3-mediated m^6^A mRNA modification has been demonstrated to play a pivotal role in terminating pluripotency and initiating differentiation in stem/progenitor cells, as well as in regulating the activation and functional competence of immune cells, such as T cells and dendritic cells (DCs). One study demonstrated that the initial 20% of m^6^A-labeled mRNA in embryonic stem cells (ESCs) exhibited enrichment in essential pathways related to embryonic development, proto-intestinal embryo formation, and cell cycles, underscoring the pivotal functions of m^6^A mRNA in cellular processes. Nevertheless, proper m^6^A mRNA modification appeared to have more beneficial effects on normal cell differentiation while showing less impact on normal cell survival or apoptosis. In METTL3 knockout (KO)-naïve mouse ESCs and pre-implantation epiblasts, m^6^A methylation on mRNA is almost entirely abolished, yet these cells remain viable. These cells fail to properly exit the naïve pluripotent state, resulting in impaired differentiation and early embryonic lethality. Mechanistically, m^6^A methylation facilitates the degradation of pluripotency-associated transcripts (e.g., Nanog, Klf4) by recruiting reader proteins such as Ythdf2. In METTL3 KO-naïve mESCs, the stability of these transcripts is significantly increased, while differentiation markers (e.g., *Gata4*, *Troponin*) are insufficiently expressed, thereby preventing naïve mouse ESCs from transitioning into the primed state [31]. Further investigation demonstrated that METTL3 depletion in m/hESC hinders ESC self-renewal both in vitro and in vivo, potentially due to elevated Nanog expression levels [27]. Targeting METTL3 in embryos was shown to impede the emergence of hematopoietic stem/progenitor cells (HSPCs) through the sustained activation of Notch signaling pathways [32]. Furthermore, m^6^A has been identified as a transcriptome flexibility marker that is essential for the differentiation of stem cells into specific lineages. Recent research has revealed its critical role in the endothelial-to-hematopoietic transition (EHT) in specifying the earliest hematopoietic stem and progenitor cells (HSPCs) [33]. In addition to its impact on early hematopoietic cells, disruptions in m^6^A methylation have been shown to significantly impede T cell homeostasis and differentiation through METTL3-mediated m^6^A mRNA catalytic activity and the regulation of the IL-7/STAT5/SOCS pathway. Naïve T cells lacking METTL3 remain in a naïve state for an extended period of 12 weeks, contributing to the resistance to colitis [34]. Furthermore, it has been demonstrated that METTL3-mediated m^6^A plays a role in promoting the maturation of dendritic cells without affecting their survival activity, which is achieved by the m^6^A-enhanced translation of CD40, CD80, and TLR4 signaling adaptor Tirap transcripts [35]. This was supported by the lack of significant differences in apoptosis observed in bone-marrow-derived dendritic cells from METTL3-WT/KO mice [35]. Additionally, m^6^A mRNA has been shown to significantly promote various normal biological functions such as adipogenesis, circadian rhythm, spermatogenesis [36,37,38], stem cell differentiation, precise spatiotemporal control of the cerebellum [39], and neurodevelopment [40].

### 3.2. Influence of m^6^A mRNA on Tumor Cell Fate

**Figure 1:**
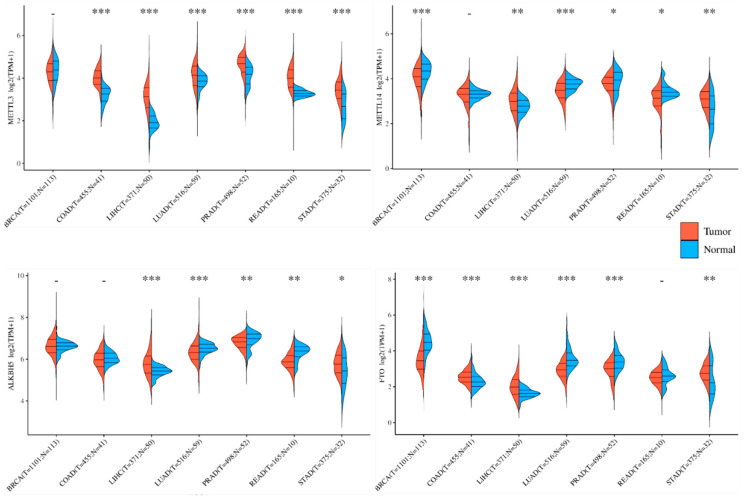
Expression of m^6^A mRNA modifiers in five common global cancers. m^6^A mRNA “writers” and “erasers” indicate substantial alterations in human tumor tissues compared to normal tissues. Here, we present their expression levels in the top five most prevalent types of tumors globally, as evidenced by data from the TCGA database. Furthermore, the inconsistent tendencies of m^6^A mRNA modifiers observed within the same type of tumors, such as variations in METTL3 vs. METTL14 in LUAD, PRAD, and READ, as well as the contrasting changes between COAD and LIHC in METTL3 expressions, underscore the need to elucidate the factors influencing m^6^A mRNA modifications in tumors. Additionally, the similar patterns observed in disparate tumor types, such as LUAD and PRAD, offer a valuable opportunity to investigate their pathogenic mechanisms and determinants. BRCA: breast-invasive carcinoma; COAD: colon adenocarcinoma; LIHC: liver hepatocellular carcinoma; LUAD: lung adenocarcinoma; PRAD: prostate adenocarcinoma; READ: rectum adenocarcinoma; STAD: stomach adenocarcinoma. * *p* < 0.05, ** *p* < 0.01, *** *p* < 0.001, - *p* > 0.05.

In tumor cells, the regulation of m^6^A methylation and demethylation is often significantly altered, impacting the overall m^6^A levels. Here, we present the expression changes of m^6^A methyltransferases METTL3 and METTL14, as well as demethylases ALKBH5 and FTO, across seven of the most prevalent cancers worldwide according to the TCGA database (Figure 1). This database shows frequently anomalous expressions of these enzymes, and METTL3 expression is always notably elevated in these tumor types. This suggests a potentially crucial role of abnormal m^6^A mRNA modification in tumor progression.

m^6^A modification is pivotal in the survival of tumor cells. In contrast to normal cells, abnormal stimulation or tumor cells exhibit distinct m^6^A mRNA methylation patterns that may play a role in survival or apoptosis. Specifically, in the context of ultraviolet-induced DNA damage, m^6^A mRNA modification by METTL3/14 has been identified as a signal for the targeted recruitment of Pol κ to damaged sites, thereby promoting DNA repair and cell survival [41]. Moreover, the acceleration of apoptosis in tumor cells, specifically HepG2 cells, can be achieved by silencing METTL3 expression. This phenomenon is likely linked to the m^6^A-modulated p53 signaling pathway [14], which plays a critical role in cell proliferation [42]. Additionally, FTO has been shown to be significantly elevated during the formation of leukemia cells, leading to an extended survival cycle and decreased cell apoptosis by upregulating oncogene expressions [43].

m^6^A methylation may promote tumor cell proliferation through various pathways. In osteosarcoma (OS), the m^6^A level for RNA methylation and the expression level of METTL3 were upregulated in human OS tissues and OS cell lines. METTL3/m^6^A significantly promotes the proliferation, migration, and invasion of OS cells through the m^6^A-mediated stability of LEF1 mRNA and the activity of Wnt/β-catenin signaling [44]. In colorectal cancer (CRC) and bladder cancer (BC), METTL3 accelerates the cell cycle of tumor cells by directly promoting the expressions of CCNE1 [45], AF4/FMR2 family member 4 (AFF4), two key regulators of the NF-κB pathway (IKBKB and RELA), and MYC [46]. In hepatoblastoma (HB), METTL3/m^6^A methylation significantly affects the Wnt/β-catenin pathway by decreasing the expression and stability of CTNNB1, the coding gene of β-catenin [47]. In hepatocellular carcinoma (HCC), METTL3 has been reported to promote tumor cell proliferation through the YTHDF2-dependent silencing of SOCS2, resulting in the overactivation of the JAK/STAT pathway [48]. Moreover, KIAA1429 has also been reported to promote the proliferation of HCC cells by promoting GATA3 degradation [49].

Besides the above basic life activities related to cell fate survival and proliferation, m^6^A methylation also plays critical roles in tumor invasion and metastasis. Multivariate Cox regression analysis revealed that METTL3 expression was an independent prognostic factor and effective predictor in human patients with gastric cancer (GC). Mechanistically, P300-mediated H3K27 acetylation activation in the promoter of METTL3 induced METTL3 transcription, which stimulated m^6^A modification and the IGF2BP3-enhanced stability of HDGF mRNA, which promoted tumor angiogenesis by activating GLUT4, ENO2 expression, and an increase in glycolysis in GC cells [50]. METTL3 can also promote the expression of transcription factor GFI-1 and α-smooth muscle actin (α-SMA) associated with EMT in GC [51]. However, in a few cases, m^6^A has been reported to inhibit tumor progression. In renal cancer (RCC), ATP-P2RX6 was reported to participate in the regulation of the Ca^2+^-mediated p-ERK1/2/MMP9 signaling pathway to enhance the migration and invasion of RCC cells, while METTL14/m^6^A signaling can downregulate the translation of the P2RX6 protein [52]. m^6^A demethylase is also suggested to play pro-tumor roles in GC, AML, and cervical cancer. In GC, ALKBH5/FTO was found to be highly expressed in tumor cells and activated Wnt and PI3K-Akt signaling to promote GC development [53]. FTO was also reported to promote tumor survival and proliferation through the inhibition of the P53/apoptotic pathway [54], and to promote E2F1 and MYC expressions [55].

The cellular state is a crucial determinant in understanding the functions of m^6^A mRNA. Cancer cells, characterized by heightened protein synthesis and dysregulated signaling pathways such as the p53 pathway, exhibit increased expression of mutant proteins involved in tumor cell apoptosis regulation. The removal of methylation enzymes may potentially result in the heightened sensitivity and aggressiveness of tumor cells compared to normal cells, although further evidence is required to substantiate this claim. A study revealed that the impact of METTL3 on the resting state of ESCs differed between the resting and activating states during the preparation for differentiation. Specifically, METTL3 was found to promote ESC differentiation and transition from a highly quiescent state in the resting state, while in the activating state, it primarily maintained their proliferation capacity and inhibited differentiation [56]. Similarly, in contrast to the beneficial effects of m^6^A in promoting normal cell differentiation, the depletion of METTL3 in AML cells resulted in a loss of self-renewal ability and an increase in differentiation capacity. This was attributed to the suppressed translation of c-MYC, BCL2, and PTEN mRNAs through m^6^A modification [56,57]. Additionally, the m^6^A-dependent negative regulation of SOCS2 has been observed in liver cancer cells [48], although a study on T cell differentiation did not find METTL3 to be responsible for the m^6^A modification of SOCS2. Thus, the involvement of m^6^A mRNA in determining cell fates may vary depending on the specific cellular state.

## 4. Tumor Cell Fate via m^6^A mRNA Targeting

### 4.1. Effects of Decreasing m^6^A

**Figure 2:**
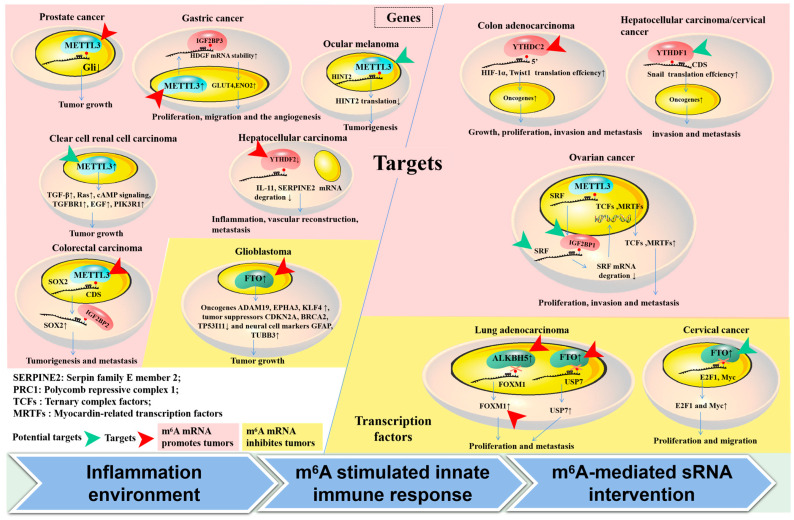
Targets involved in m^6^A mRNA methylation in tumors. (1) The methylation of m^6^A mRNA has been shown to regulate gene expression through the direct modification of executive function genes (**left** part) and transcription factors (**right** part) such as HIF-1α, Snail, and FOXM1, impacting tumor status. (2) Valid targets for m^6^A mRNA regulators and their target genes, as demonstrated in animal models, are highlighted with red arrows. Potential targets are also suggested (indicated by a green arrow). The distinct effects of m^6^A mRNA modification on tumors are distinguished, both promoting (pink region) and inhibiting (yellow region). (3) Three factors are enumerated in the lower portion of the diagram that could potentially impact variations in effectiveness across various tumors following the m^6^A targeting of the same gene or the same m^6^A-associated regulators.

Numerous essential pathways have been documented as being directly or indirectly influenced by m^6^A mRNA in the progression of tumors (Figure 2); nevertheless, numerous signaling pathways exhibit inconsistent regulation or even diametrically opposed regulation across various tumor types. In this study, we delineated several potential therapeutic targets for m^6^A-related tumors, encompassing signaling pathways and m^6^A regulators that are ubiquitously present in multiple tumor types.

The Wnt/β-catenin pathway, which plays a significant role in regulating gene transcription by utilizing β-catenin as a transcription co-activator in conjunction with the LEF family, has been found to be a key factor in promoting epithelial–mesenchymal transition (EMT). A recent study on osteosarcoma (OS) revealed that the inhibition of METTL3 can disrupt Wnt signaling activity by altering the m^6^A levels of LEF1, resulting in the suppression of proliferation, migration, and invasion in OS cells [44]. In hepatoblastoma (HB), the knockdown of METTL3 notably decreased the expression and stability of CTNNB1 mRNA, which encodes β-catenin that binds to the TCF4/LEF1 transcription factor and activates target genes such as Jun, MYC, and CCND1, resulting in significant inhibition of HB cell proliferation and colony formation [47]. Further, the overexpression of ALKBH5 significantly inhibited the proliferation, migration, and invasion of pancreatic ductal adenocarcinoma (PDAC) cells by inhibiting the transactivation of Wnt inhibitory factor 1 (WIF-1) [58]. Hence, it is proposed that targeting Wnt signaling pathways by reducing m^6^A methylation may serve as an effective strategy for treating tumors.

Moreover, several key genes involved in cell proliferation and tumor cell survival have been shown to be negatively regulated by decreasing m^6^A mRNA in various types of tumors. For example, BCL2 and MYC, well-known oncogenes that inhibit apoptosis and promote cell proliferation, have been reported to undergo m^6^A-mediated translation, stabilization, or indirect promotion in bladder cancer [46,59], lung cancer [60], breast cancer [61,62], acute myeloid leukemia (AML) [63], and gastric cancer [64,65]. Moreover, decreasing m^6^A has demonstrated potential therapeutic efficacy through the regulation of the YAP/CCNE1, HDGF, and cAMP pathways, which are commonly associated with tumor proliferation. CCNE1, a nucleoprotein that is expressed cyclically, plays a crucial role in the mitosis of eukaryotic cells by activating CDK2 and forming a complex to traverse the G1/S phase checkpoint [66]. CCNE1 overexpression leads to chromosome instability and enhances the proliferation and metastasis of tumor cells. In CRC, the downregulation of METTL3 exhibited significantly decreased expression levels and weakened the mRNA stability of CCNE1. Notably, it has been documented that CCNE1 is positively controlled by YAP1 through the formation of a complex with TEAD4 in metformin-attenuated cancer proliferation [67]. Furthermore, the YAP signaling pathway has been shown to be subject to m^6^A-mediated regulation through the knockdown of METTL3, which directly enhances YAP translation by recruiting YTHDF1/3 and eIF3b to the translation initiation complex [68]. Additionally, ALKBH5 has been reported to inhibit YAP activity by regulating the miR-107/LATS2 axis in a HuR-dependent manner [68].

In addition, the activation of the HDGF-PI3K-AKT-mTOR axis could also be inhibited after decreasing m^6^A through the disrupted Circ-CDYL biogenesis mediated by m^6^A/YTHDC1 and the m^6^A/hnRNPA2/B1-mediated active sorting of Circ-CDYL into exosomes, ultimately attenuating tumor malignancy [69]. Consequently, targeting the m^6^A of the core genes essential for the survival and proliferation of tumor cells shows promise as a potential therapeutic strategy.

In addition to the aforementioned points, the potential efficacy of targeting m^6^A methylation as a treatment for tumors is further supported by its direct regulation of multiple transcription factors. It was discovered that decreasing m^6^A inhibited the expression of SRF, which controls key gene classes such as TCFs and MRTFs, through the mRNA-binding protein IGF2BP1. The depletion of Insulin-like growth factor 2 mRNA-binding protein 1 (IGF2BP1) can accelerate the degradation of SRF directed by miRNA. This alteration was found to be particularly significant in ES-2 cells when compared to other cancer cell lines such as Huh-7 cells [70]. The downregulation of METTL3 can significantly restrain liver metastasis in gastric cancer (GC) mouse models through the disruption of the m^6^A reader IGF2BP3-mediated enhancement of hepatoma-derived growth factor (HDGF) transcript stability, which plays a crucial role in tumor cell proliferation, migration, and angiogenesis [50]. Another significant driver of tumor growth, HIF-1, has been shown to target approximately 50 genes and play a crucial role in various aspects of tumor progression, including proliferation, invasion, and metastasis. Recent findings indicate that alterations in the m^6^A modification of the HIF-1 signaling pathway are notably present in samples from patients with clear-cell renal cell carcinoma (ccRCC). Additionally, targeting YTHDC2 has been demonstrated to reduce the translation efficiency of HIF-1α, dependent on the 5′ UTR in hypoxic conditions, thereby suppressing metastasis in colon adenocarcinoma [71]. The findings indicate a significant positive correlation between YTHDC2 expression levels and tumor stage in colon cancer patients, underscoring the pivotal role of YTHDC2 in cancer progression.

Furthermore, the upregulation of cancer suppressor genes through decreasing METTL3/m^6^A has also been shown to effectively inhibit tumor growth. For instance, METTL3 has been reported to negatively regulate the cancer suppressor gene GIi, a crucial component of the hedgehog pathway, thereby promoting PC growth. The downregulation of METTL3 can significantly increase the expression of Gli and limit cancer growth [72]. Additionally, m^6^A-dependent negative regulations of TGF-β, Ras signaling pathways, and the tumor suppressor genes HINT2 and SOCS2 have also been observed in ocular melanoma, liver cancer, and other related malignancies [48,73]. Thus, decreasing m^6^A is potentially implicated in tumor therapy by downregulating oncogene expression and upregulating tumor suppressor genes in various key pathways, including Wnt/β-catenin, proliferation/survival via direct mRNA regulation, and transcription factors such as HIF-1, CHX8, and SRF.

### 4.2. Effects of Increasing m^6^A

While m^6^A methylation has been demonstrated to be carcinogenic in many tumors, the demethylation of m^6^A and its regulators, such as ALKBH5 and FTO, have also been shown to contribute to carcinogenesis. Additionally, increasing m^6^A methylation has been found to inhibit tumor progression in specific tumor types, including glioblastoma, lung cancer, cervical cancer, and endometrial tumors. In the context of glioblastoma, the inhibition of FTO rather than METTL3 or METTL14 was shown to inhibit the proliferation of glioblastoma stem cells (GSCs) and tumorigenesis through the modulation of various oncogenes (ADAM19, EPHA3, and KLF4), tumor suppressors (CDKN2A, BRCA2, and TP53I11), and neural cell markers (GFAP, TUBB3) [74]. FOXM1, a member of the Fox gene family, plays a critical role as a transcription factor in cell division, tumor progression, and distant metastasis [75]. The downregulation of ALKBH5 has been identified as a significant method for restraining the proliferation and malignancy of lung adenocarcinoma cells through decreasing the translation efficiency of FOXM1 mediated by m^6^A modification [76]. The deletion of ALKBH5 has also been shown to suppress the growth and invasion of lung adenocarcinoma cells. Furthermore, the downregulation of FTO in lung cancer inhibited the growth of cancer cells both in vitro and in vivo through the repression of USP7 expression in an m^6^A-dependent manner [77]. Similarly, the downregulation of FTO has been shown to inhibit the proliferation and migration of cervical cancer cells (HeLa and SiHa) by reducing the translation efficiency of E2F1 and MYC transcripts in an m^6^A-dependent manner [55].

Some evidence of an association between reduced m^6^A and poor cancer outcomes has also been reported. In endometrial tumors, approximately 70% of patients demonstrate decreased rather than increased m^6^A methylation, likely due to the presence of the METTL14 (R298P) mutation or the reduced expression of METTL3. This reduction in m^6^A methylation has been shown to enhance the proliferation and migration of endometrial cancer cells [78,79]. Additionally, the downregulation of PHLPP2 and upregulation of mTORC2, which respectively serve as negative and positive regulators of AKT, have been identified as key mediators in these tumor types [78]. Consequently, increasing m^6^A demethylation may represent a more promising therapeutic approach for these tumors.

### 4.3. Determiners of Targeting m^6^A mRNA in Tumors

As depicted in Figure 2, the presence of m^6^A has been shown to elicit a positive response to external stimuli through intricate regulatory mechanisms. However, it is important to note that these responses vary depending on the specific inflammatory or environmental conditions present within tumors. For example, the exposure of HEPG2 cells to UV or heat shock resulted in alterations to the 5′ UTR m^6^A metagene profile, whereas treatment with IFN-γ stress did not have the same effect, highlighting the unique characteristics of 5′ UTR m^6^A in response to stress. Additionally, environmental factors within tumors, such as hypoxia and lactic acid, have been shown to impact m^6^A mRNA modifications by altering the expressions of m^6^A regulators or impacting their functions. For instance, HIF-1α has been shown to directly stimulate the transcription of YTHDF1, thereby facilitating hepatocellular carcinoma (HCC) autophagy and malignancy by enhancing the translation of ATG2A and ATG14 [80]. In breast cancer cells, ALKBH5 has been reported to exhibit HIF-1α- and HIF-2α-dependent expression [81]. Additionally, HIF-1α has been implicated in promoting the expression of WTAP, which, in turn, mediates the expression of the glycolysis enzyme HK2 to enhance the Warburg effect in ovarian cancer [82]. Furthermore, HIF-1α has been found to promote METTL3 expression in non-small-cell lung cancer (NSCLC) under smoking-induced stimulation. Furthermore, it has been documented that HIF-1α can impede m^6^A-mediated mRNA regulation by exerting competitive influences [83]. Specifically, HIF-1α has the ability to enhance the expression of LncRNA STEAP3-AS1, leading to its competitive interaction with YTHDF2. This interaction serves to shield STEAP3 mRNA from YTHDF2/m^6^A-mediated degradation, thereby facilitating the progression of colorectal cancer [84]. Additionally, lactic acid may serve as a significant environmental factor influencing m^6^A modification. Studies have shown that the accumulation of lactate in the tumor microenvironment can induce the upregulation of METTL3 in tumor-infiltrating immune cells through H3K18 lactylation, a process crucial for METTL3 to bind to target RNA [85]. Furthermore, the lactylation of METTL16 has been found to promote cuproptosis via the m^6^A modification of FDX1 mRNA in gastric cancer. Additionally, the lactylation of histones has been implicated in oncogenesis by enhancing the expression of the m^6^A reader protein YTHDF2 in ocular melanoma [86]. The upregulation of lactate-induced H3K18 lactylation has also been reported to promote the transcription of YTHDF1 [87]. It is known that the degree of hypoxia/HIF-1 or lactate content within the tumor microenvironment is obviously different in different tumor types or tumor stages, which may lead to differences in the expression of m^6^A mRNA modification regulators and real-time changes, as well as differences in the therapeutic efficacy of targeted m^6^A. Therefore, all of these reports suggest that we should pay attention to not only the downstream mechanism of m^6^A regulation, but also the effect of targeted m^6^A therapy. The consideration of upstream environmental factors influencing m^6^A modification is essential for optimizing the effectiveness of targeted m^6^A therapy and adjusting dosages for personalized treatment. Additionally, investigating the specific triggers of m^6^A mRNA modification can enhance the understanding of pathogenesis, the dual roles potentially associated with various tumor progressions, and the development of combination immunotherapy approaches for tumors.

Additionally, the intricate regulatory mechanisms involving m^6^A modification play a crucial role in determining the targeting of m^6^A-modified mRNA in tumors. This includes factors such as disrupted mRNA binding with HuR and the impact of non-coding RNAs. On the one hand, it became evident over time that m^6^A modification in mRNA impedes the binding of the RNA-binding protein HuR, leading to the accelerated degradation of targeted mRNAs. For example, the upregulation of KIAA1429 has been shown to drive tumor cell proliferation and metastasis, correlating with unfavorable outcomes in hepatocellular carcinoma (HCC) patients. This upregulation can result in m^6^A methylation on the 3′UTR of GATA3, a transcription factor known for its role as a potent tumor suppressor involved in the growth and differentiation of malignant cells and anti-tumor immune regulation, ultimately leading to the enhanced degradation of GATA3 [88,89]. The m^6^A modification on the GATA3 3′UTR mechanistically initiates the dissociation of HuR from GATA3 pre-mRNA [49] in a sequential manner. Additionally, the impediment of m^6^A in mRNA from interacting with HuR was corroborated in a separate investigation of hepatocellular carcinoma, where WTAP-mediated m^6^A modification in ETS1 led to its dissociation from HuR, hastening its degradation and promoting the advancement of hepatocellular carcinoma [90]. On the other hand, it was noteworthy that the long non-coding RNA GATA3-AS, transcribed from the antisense strand of the GATA3 gene, served as a cis-acting element facilitating the preferential interaction of KIAA1429 with GATA3 pre-mRNA. This mechanism, which involves the collaboration of its homologous LncRNA and m^6^A regulators, aligns closely with findings in ALKBH5-mediated demethylation. Specifically, the role of LncFOXM1-AS in promoting the interaction between ALKBH5 and FOXM1 nascent transcripts, thereby contributing to the maintenance of tumorigenicity in glioblastoma stem-like cells, suggests a novel m^6^A regulatory pathway by non-coding RNA [91]. Hence, it is evident that diverse m^6^A regulation patterns are present in tumor cells and their microenvironments, necessitating the utilization of advanced technical approaches, such as organoids, to comprehensively assess the effects of targeted m^6^A modifications on tumors from various perspectives and mitigate the risk of obtaining inaccurate findings.

## 5. Contributions of m^6^A Non-Coding RNAs in Tumors

Non-coding RNAs, such as miRNA, LncRNA, and circRNA, are a significant group of RNA molecules that primarily regulate gene expression at the post-transcriptional level. These non-coding RNAs play a crucial role in fundamental biological processes such as cell growth, development, metabolism, and tumor progression. Recent research has indicated that the expression of non-coding RNAs is influenced by m^6^A modification. Furthermore, m^6^A has been suggested to act as a mediator between mRNA and non-coding RNAs, such as miRNA, in modulating specific cellular functions [92,93].

In normal cells, m^6^A modification likely enhances the processing of miRNA/LncRNA. The splicing of pri-miRNA requires the involvement of the miRNA microprocessor complex, which consists of the RNA-binding protein DGCR8 and the type III RNase DROSHA. It has been observed that m^6^A-modified pri-miRNAs can interact with DGCR8 and recruit DROSHA, thereby expediting the splicing of pri-miRNA. The depletion of METTL3 has been shown to decrease the binding of DGCR8 to pri-miRNAs, leading to a global reduction in mature miRNAs and the accumulation of unprocessed pri-miRNAs [94]. Furthermore, hnRNPA2B1 has the capability to recognize m^6^A modifications, directly interact with m^6^A marks on a subset of pri-miRNA transcripts, and engage with DGCR8 to facilitate the splicing process of pri-miRNAs. It has been observed that hnRNPA2B1 binds to a specific set of nuclear transcripts and induces alternative splicing effects similar to those of the m^6^A writer METTL3 [95]. Additionally, the long non-coding RNA X-inactive specific transcript (LncXIST) is responsible for mediating the transcriptional silencing of genes on the X chromosome and plays a crucial role in female mammalian development. In 2016, a study demonstrated that LncXIST contains a minimum of 78 m^6^A residues, with RBM15/RBM15B facilitating its function by facilitating the interaction between WTAP–METTL3 and LncXIST. Additionally, YTHDC1 was found to selectively recognize m^6^A residues on LncXIST and was essential for its function [15], highlighting the role of m^6^A as a regulator of transcriptional repression by long non-coding RNA.

**Figure 3:**
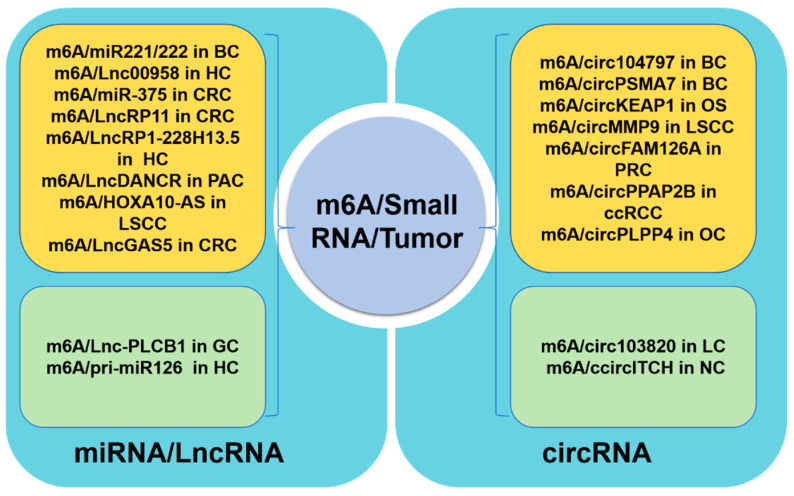
Roles of m^6^A methylation on small RNA in tumor cells. The regulation of tumor progression in tumor cells by m^6^A RNA is partially mediated through the modulation of miRNA/LncRNA (the **left** part) or circRNA (the **right** part). Specifically, the yellow region signifies a positive regulation of tumor progression by m^6^A, while the green region denotes a negative regulation of tumor progression. BC: bladder cancer; HC: hepatocellular carcinoma; CRC: colorectal cancer; PAC: pancreatic cancer; LSCC: laryngeal squamous cell carcinoma; GC: gastric cancer; OS: osteosarcoma; PRC: prostate cancer; ccRCC: clear-cell renal cell carcinoma; OC: ovarian cancer; LC: lung cancer; NC: nasopharyngeal carcinoma.

In tumor cells, m^6^A modifications on miRNA/LncRNAs have been shown to promote tumor pathogenesis, as illustrated in Figure 3. For instance, in bladder cancer, the maturation of pri-miR221/222 has been found to be expedited by METTL3-mediated m^6^A modification through its interaction with DGCR8, resulting in decreased PTEN expression [59]. Furthermore, in hepatocellular carcinoma cells, a lipogenesis-related LncRNA known as Lnc00958 was identified as being positively regulated by m^6^A. This LncRNA was found to act as a sponge for miR3619-5p, leading to the upregulation of HDGF expression and ultimately promoting tumor lipogenesis and progression [96]. Additionally, the progression of liver cancer was shown to be facilitated by the m^6^A methyltransferase METTL14-mediated RP1-228H13.5, which targets hsa-miR-205/ZIK1 [97]. In colorectal cancer tissues, the downregulation of METTL14 resulted in reduced levels of miR-375, leading to the promotion of CRC growth and migration through the YAP1 and SP1 pathways, respectively. Based on additional research, it has been determined that the m^6^A-mediated regulation of the YAP pathway involves not only miRNA, but also direct interactions with LncRNA. Specifically, a negative feedback loop involving the LncRNA GAS5-YAP-YTHDF3 axis has been identified, wherein LncGAS5 promotes YAP degradation through ubiquitination and intracellular translocation, while YAP upregulates YTHDF3 expression, leading to the degradation of m^6^A-modified LncGAS5 and ultimately inhibiting YAP degradation, thereby contributing to the progression of colorectal cancer [98]. A recent study revealed that a novel LncRNA, RP11, plays a positive regulatory role in the migration, invasion, EMT, and liver metastasis of CRC by forming an RP11/hnRNPA2B1/mRNA complex, which is dependent on m^6^A-mediated upregulation [99]. Additionally, in pancreatic cancer, LncDANCR has been identified as a novel target for reader IGF2BP2 through m^6^A modification, leading to the stabilization of LncDANCR and the promotion of cancer proliferation and stem-like properties [100]. The m^6^A/HOXA10-AS/ITGA6 axis contributes to increased oxidative resistance and facilitates the malignant advancement of laryngeal squamous cell carcinoma through the modulation of the Notch and Keap1/Nrf2 pathways [101]. These studies demonstrate the significant impact of m^6^A modification on tumor progression via the regulation of miRNA and LncRNA expression, underscoring the promising prospects for targeted m^6^A RNA therapy.

circRNA, functioning as a molecular sponge for microRNA (miRNA), modulates gene expression within signaling pathways, thereby influencing tumor cell proliferation, migration, invasion, apoptosis, and drug resistance. This characteristic renders circRNA a promising target for therapeutic interventions in cancer. The regulation of circular RNAs through m^6^A modification has recently been extensively proposed [102], with evidence suggesting that the upregulation of m^6^A-mediated circRNA expression is linked to the progression of various types of tumors including bladder cancer, clear-cell renal cell carcinoma (ccRCC), osteosarcoma, laryngeal squamous cell carcinoma, nasopharyngeal carcinoma, prostate cancer, and ovarian cancer [103]. For instance, research has demonstrated that elevated levels of m^6^A in circ_104797 [104] and circPSMA7 [105] play a crucial role in maintaining cisplatin resistance and advancing bladder cancer progression. Additionally, the m^6^A modification on circPPAP2B modulates the interaction between HNRNPC and the splicing factors PTBP1 and HNPNPK, thereby regulating pre-mRNA alternative splicing in ccRCC [106]. In osteosarcoma, the decreased expression of m^6^A-mediated circKEAP1 promotes the proliferation and invasion of tumor cells. In the context of lung cancer, the suppression of hsa_circRNA_103820 expression by IGF2BP3/m^6^A has been observed to augment the migratory and invasive properties of cancer cells [107]. Additionally, the stabilization of CircMMP9 by IGF2BP2/m^6^A has been shown to expedite the progression of laryngeal squamous cell carcinoma by facilitating TRIM59 transcription [108]. Furthermore, the downregulation of circITCH mediated by HNRNPC has been implicated in the pathogenesis of nasopharyngeal carcinoma by impeding the sequestration of miR-224-3p [109]. Lastly, the m^6^A modification of circFAM126A has been demonstrated to enhance transcriptional stability and promote the progression of prostate cancer [110]. Moreover, a recent study identified a new interaction between circular RNA (circRNA) and m^6^A reader proteins, demonstrating a regulatory function in m^6^A modifications. Specifically, circRARS has been shown to cooperate with IGF2BP3 to increase m^6^A recognition, thereby promoting the advancement of renal cell carcinoma [111].

Hence, the ability of non-coding RNA to modulate the expression of numerous genes—potentially hundreds—represents a distinctive benefit of non-coding RNA in the context of anti-tumor therapy. It is increasingly evident that m^6^A modification is pivotal in driving tumor advancement by controlling the expression of a significant array of non-coding RNAs. Manipulating the expression of non-coding RNAs via m^6^A modification to impact multiple downstream pathways, thereby reshaping and redefining the tumor microenvironment, emerges as a highly promising avenue for further research. Further investigation into the impact of m^6^A modifications on non-coding RNAs in tumor-related processes has the potential to inform novel therapeutic approaches targeting m^6^A in tumor treatment strategies.

## 6. Perspectives on m^6^A RNA Targeting for Tumor Treatment

### 6.1. Strategies for Targeting m^6^A mRNA in Tumor Therapy

**Figure 4:**
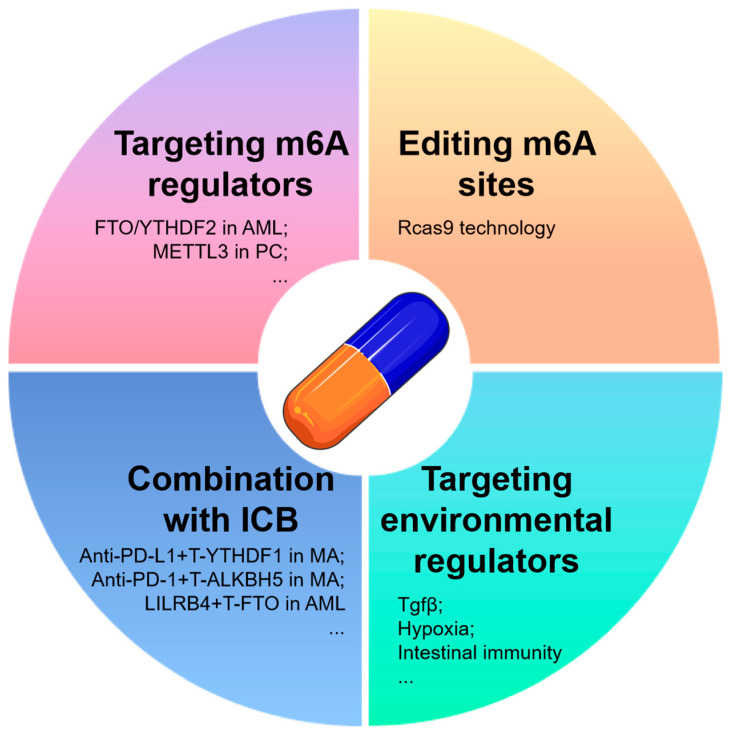
Therapy strategies targeting m^6^A RNA in tumors. Here, we present four tumor treatment strategies focused on targeting m^6^A RNA: targeted m^6^A RNA regulators, the utilization of Cas9 or other advanced technologies for the direct manipulation of gene m^6^A targets, combination therapy with traditional tumor treatments or tumor immune checkpoint (ICB) inhibitors to enhance therapeutic efficacy, and the targeted modulation of m^6^A-related tumor microenvironmental factors. PC, pancreatic cancer; ICB, immune checkpoint blockade; T-, targeting; MA, melanoma.

According to reports, the silencing of METTL3 in HepG2 cells resulted in a significant enrichment of the p53 signaling pathway, with 22 out of 23 genes showing differentially expressed splice variants, 18 of which were found to be m^6^A-methylated. Table 2 demonstrates the significant regulation of m^6^A mRNA methylation in tumor formation. Here, we propose four strategies for targeting m^6^A mRNA in tumor therapy (Figure 4). The first strategy involves targeting m^6^A regulators, such as FTO or YTHDF2, which have been identified as potential adjuvant therapeutic targets in AMLs. These targets have shown promise in effectively regulating the proliferation and life cycle of leukemic cells, as well as enhancing ATRA-induced differentiation [43,112]. Numerous studies have demonstrated the potential efficacy of targeting m^6^A regulators, specifically METTL3, for improving the treatment of gemcitabine in pancreatic cancer [113]. Additionally, R-2HG has been shown to possess anti-tumor activity through its targeting of the FTO/m^6^A/MYC/CEBPA signaling pathway [114]. However, due to the complex regulatory mechanisms and dual roles of m^6^A mRNA regulators in certain tumors, the therapeutic effectiveness of strategies aimed at inhibiting or promoting methylase expressions may be constrained or unpredictable. Hence, the integration of this approach with siRNA-based biomedical engineering holds promise for enhancing therapeutic outcomes and may lead to the exploration of more precise methods for tumor treatment as potential avenues for further research. In the second approach, the modification of m^6^A sites through RCas9 technology [115] may be utilized to precisely alter the m^6^A sites of specific targets. The implementation of such procedures is reliant on advancements in tumor-targeting technology.

Disrupting the mechanisms that link environmental factors to m^6^A alterations could potentially have comparable effects across various cancer types, including, but not limited to, TGF-β, intestinal immunity, heat shock [118], lactate acid, and hypoxia [76]. Research shows that m6A is a dynamic event directly modulated by extracellular signals such as TGFβ. In hPSCs, SMAD2/3 binds with METTL3/METTL14/WTAP in a TGFβ-dependent Activin/Nodal signaling manner, promoting the m^6^A modification of pluripotency genes (e.g., NANOG, Nodal, LEFTY1) near the termination codon, inducing their degradation and prompting the cells to exit pluripotency [119]. Additionally, TGF-β promotes METTL3 liquid–liquid phase separation, reducing ITIH1 mRNA stability and advancing HCC progression [120]. As a secreted protein, ITIH1 acts as a ligand for the integrin α5β1, antagonizing fibronectin, inhibiting the focal adhesion kinase pathway, and suppressing HCC progression. In preclinical models (mouse, patient-derived organoids, patient-derived xenografts), purified recombinant ITIH1 (r-ITIH1) protein targets HCC and synergizes with TGF-β inhibitors in therapy [120]. ALKBH5 promotes FOXA1 protein expression by inhibiting m^6^A modification in its CDS region, while TGF-β1 reduces ALKBH5’s binding to the FOXA1 CDS, increasing m^6^A modification and suppressing FOXA1 expression, thereby promoting GBC EMT and metastasis [121]. Thus, TGFβ, as a key regulator of m^6^A modification and tumor metastasis, may potentially enhance m^6^A-targeted therapies when inhibited.

Research shows that butyrate, a classic intestinal microbial metabolite, downregulates METTL3 and cyclin E1 expression to inhibit CRC development, highlighting a potential strategy for intervening in m^6^A modification by regulating gut microbial metabolism in colorectal cancer [45]. As described in Section 4.3, hypoxia-triggered HIF-1α in the tumor microenvironment promotes tumor progression by enhancing the expression of m^6^A regulators (e.g., YTHDF1, WTAP, METTL3) and altering their functions (e.g., competitively inhibiting YTHDF2 recognition). Lactate also promotes tumor progression by regulating the expression and function of m^6^A factors such as METTL3, YTHDF2, and METTL16. Therefore, these m^6^A-regulated factors may serve as novel targets for cancer diagnosis and the development of therapeutic interventions. Investigating the specific triggers of m^6^A mRNA modification can enhance the understanding of pathogenesis, the dual roles potentially associated with various tumor progressions, and the development of combination immunotherapy approaches for tumors.

In the field of clinical oncology, monotherapy using chemical agents is often ineffective due to the emergence of drug resistance. The study of cancer biology is intricately linked to epigenomic regulation. In recent years, there has been significant progress in the development of therapies targeting epigenetic mechanisms, exemplified by the use of DNA methyltransferase inhibitors such as azacytidine and decitabine, as well as histone deacetylase inhibitors. The integration of conventional tumor therapy with novel epigenetic medications represents a promising avenue for the development of anticancer drugs in the future. The involvement of m^6^A mRNA regulators in the resistance to tumor radiotherapy, kinase inhibitors (KIs), 5-fluorouracil (5-FU), gemcitabine, anthracyclines, and cisplatin has demonstrated significant clinical utility [122,123]. Furthermore, the combination of m^6^A mRNA regulation with immune checkpoint blockade (ICB) has emerged as a viable strategy for the treatment of tumors. According to reports, the deletion of YTHDF1 in conjunction with PD-L1 checkpoint blockade has been shown to significantly improve the therapeutic effectiveness in melanoma mouse models [124]. Additionally, ALKBH5, but not FTO, has been implicated in having a detrimental impact on the response to anti-PD-1 therapy in both melanoma mouse models and patients [125]. The removal of ALKBH5 in melanoma cell lines may impact lactate levels and the presence of immunosuppressive cells in the tumor microenvironment, such as regulatory T cells (Tregs) and myeloid-derived suppressor cells (MDSCs), through m^6^A-mediated splicing regulation. This alteration could significantly improve the efficacy of the melanoma response to GVAX/anti-PD-1 treatment, which has also demonstrated effectiveness in a colorectal cancer model during anti-PD-1 therapy [125]. Furthermore, the attenuation of leukemia by suppressing immune checkpoint genes, particularly LILRB4, has been proposed to exhibit enhanced efficacy with the assistance of genetic depletion or the inhibition of FTO. This effect is attributed to the restriction of stem cell self-renewal and the reprogramming of the immune response [126]. Additionally, decreased expression of ALKBH5, METTL3, HNRNPC, and KIAA1429 in lung squamous cell carcinoma (LUSC) has been identified as a potential biomarker for increased sensitivity to immunotherapy and chemotherapy [127], suggesting potential targets for tumor treatment. Hence, anticipating the potential efficacy of combining immunotherapy with epigenetic therapy is warranted. Additionally, the consideration of combining multiple drugs targeting either similar or different types of epigenetic modifications for neoadjuvant therapy should be taken into account, despite the potential limitations observed in certain combinations such as the reduced effectiveness seen with the combination of DNMTi and HDAC inhibitors in myelodysplastic syndrome. The identification of new therapeutic targets and ongoing clinical trials are essential for the development of more effective therapy combinations in the evolving landscape of epigenetic molecular mechanisms.

An additional approach to selectively targeting m^6^A involves the utilization of RNA drugs, which have demonstrated efficacy in diverse therapeutic contexts. The successful creation and widespread administration of the COVID-19 mRNA vaccine has enhanced confidence in the practicality of RNA drugs for clinical applications. While RNA drugs show potential for exploration by emerging researchers, it is important to note that RNA can also trigger an innate immune response similar to mammalian DNA [128] through the activation of the TLR family [129]. The extent of this impact is closely linked to the specific type and quantity of RNA modifications present [130]. The activation of Toll-like receptors 7 and 8 by RNA-containing m^6^A and s2U modifications and Toll-like receptor 3 by RNA-containing m5C, m5U, or Ψ modifications has been documented. Unmodified RNA has the ability to activate all human Toll-like receptors. Therefore, aside from the challenges associated with delivering RNA drugs to specific anatomical sites and the endogenous cellular mechanisms involved in protein modifications derived from RNA drugs, the RNA-induced innate immune response is recognized as a key factor in determining tumor outcomes. The presence of m^6^A modification may prove to be advantageous in this context. Research has indicated that the presence of m^6^A in RNA can diminish or abolish the activation of human DCs triggered by RNA. This apparent inverse correlation between RNA modification and immune activation may elucidate the mechanism by which RNA from necrotic cells can stimulate DCs to secrete IFN-α, while RNA from apoptotic cells cannot, underscoring the importance of RNA degradation during apoptosis [131]. The presence of m^6^A modifications in viral mRNAs, with up to eight modifications per 1.8 kb segment of influenza RNA, has been shown to effectively inhibit the ability of RNA to activate dendritic cells. Consequently, the potential benefits of incorporating m^6^A modifications or other RNA modifications to enhance drug stability should be carefully considered when developing RNA-based therapies for tumors.

In the realm of comprehending the mechanisms of m^6^A RNA, artificial intelligence shows promise in facilitating the prediction of RNA structures and RNA–protein interactions. Nevertheless, the efficacy of machine learning algorithms is contingent upon the availability of comprehensive training data sets, which are presently more prevalent for proteins than for RNA. Moreover, artificial intelligence technology can be harnessed to pinpoint potential therapeutic targets and develop customized coding RNA drugs aimed at specific proteins. Additionally, endeavors can be undertaken to forecast m^6^A RNA splicing patterns based on genomic sequences. Several predictive models have been developed to forecast patterns of gene expression by analyzing chromatin modification [132]. While some predictive patterns for cancer prognosis based on m^6^A mRNA levels or regulators have been proposed, there is potential for the discovery of more reliable models to predict gene expression patterns using m^6^A mRNA modification. This study posits that the validity of results and conclusions derived from animal models must be extrapolated to human patients. The potential implications of this extrapolation include advancements in the manipulation and regulation of gene expression levels, as well as the study and treatment of cancers associated with specific gene expressions, potentially leading to personalized therapeutic approaches.

### 6.2. Clinical Trials and Drug Development for Tumor Therapy Targeting m^6^A

Currently, clinical trials for drugs targeting m^6^A regulators in cancer therapy are still in the early stages, and no anticancer drugs targeting m^6^A have been approved by the FDA. However, several drugs targeting m^6^A regulators have been developed and are undergoing clinical research for cancer treatment. It was reported that multiple AML cell lines and PDX models exhibited sensitivity to METTL3 inhibition by STC-15. METTL3 inhibition resulted in the dose-dependent downregulation of the anti-apoptotic protein BCL-2. A matrix combination experiment demonstrated strong synergy between STC-15 and venetoclax, a BCL-2 inhibitor, in MOLM-13 cells and an AML PDX model, significantly extending animal survival. At present, a highly selective METTL3 inhibitor, STC-15, has been developed as a specific inhibitor of m^6^A methylase METTL3 for stage I clinical use in malignant tumors whose indications include lung/hepatic/bone/colorectal/ skin/mammary gland cancer and malignant melanoma (NCT05584111, Table 3).

Two small-molecule compounds (CS1 and CS2; CS1 also means Bisantrene) which specifically target FTO and inhibit its m^6^A demethylase activity have been identified through in silico screening and validation. CS1 and CS2 bind to the catalytic pocket of FTO, disrupting its interaction with m^6^A-modified RNAs. Both compounds significantly reduced the viability and growth of human AML cells, with IC50 values in the low nanomolar range, at least 10-fold more potent than previous FTO inhibitors (e.g., FB23-2 and MO-I-500). Mechanistically, CS1 and CS2 promote anti-leukemic effects by inhibiting FTO activity, activating apoptosis signaling, and suppressing MYC pathways [126]. Targets of one clinical trial involving CS1 for the treatment of relapsed or refractory AML by combination therapy with fludarabine and clofarabine have entered Phase II (NCT04989335). In addition, the combined vaccine of IGFBP2, HER2, and IGF-1R and a small-molecule inhibitor, AST-201, have also entered clinical trials for AML and breast cancer therapy, respectively. Some other drugs have also entered the preclinical stage, such as a METTL3 inhibitor, next-generation CS1 targeting FTO, and EP-102 targeting METTL3 in malignant tumors. We list some emerging drugs targeting m^6^A that have not yet entered clinical trials in Table 4.

### 6.3. Challenges in the Development of Drugs Targeting m^6^A for Tumor Therapy

Targeting m^6^A with small-molecule inhibitors, RNA drugs, and other therapeutic approaches holds great promise in modulating gene expression and controlling various biological processes. Targeting m^6^A (N^6^-methyladenosine) in cancer therapy presents several challenges that need to be addressed for its effective clinical application.

First, these therapies may cause off-target effects, which refer to unintended interactions with molecules other than the intended targets, potentially leading to adverse biological responses and affecting drug efficacy. Small-molecule inhibitors that target m^6^A-associated enzymes, such as METTL3, METTL14, and FTO, may interact with other enzymes or molecular pathways with similar structures, resulting in the unintended modulation of RNA modification processes or other metabolic pathways, thus inducing off-target effects. RNA drugs, such as mRNA, siRNA, or antisense oligonucleotides (ASOs), may bind to non-target RNAs, disrupting cellular function and gene expression balance. Moreover, external factors such as TGFβ in the tumor microenvironment may also interfere with m^6^A regulation, further complicating the effects of these drugs.

To improve the selectivity and targeting of m^6^A-based drugs, significant advancements must be made in drug design and molecular optimization. By refining the molecular structure and increasing the affinity of drugs for specific m^6^A-related enzymes or RNA sequences, the possibility of unintended interactions with non-target molecules can be minimized. Utilizing computational drug design tools, such as computer-aided drug design (CADD), could help predict the binding affinity of drug candidates for both target and off-target molecules, guiding the development of more specific therapies. Additionally, RNA drugs can be designed to precisely recognize and regulate specific mRNA targets, reducing the likelihood of interactions with non-target RNAs. Integrating gene-editing technologies, such as CRISPR/Cas, can also offer a more precise approach to m^6^A modification, further improving therapeutic specificity.

Optimizing drug delivery systems is another promising avenue for reducing off-target effects. Techniques such as nanoparticle carriers, liposomes, and antibody–drug conjugates (ADCs) can enhance the accumulation of drugs in target tissues or cells, minimizing their impact on non-target areas. By incorporating receptor-targeted and cell-penetrating strategies, drugs can be directed specifically to the affected regions, improving therapeutic outcomes while reducing side effects. Moreover, advances in precision medicine and personalized therapies, supported by genomic, transcriptomic, and single-cell technologies, could enable tailored treatments based on individual genetic profiles. By identifying patient-specific variations in m^6^A regulation, personalized therapeutic approaches can be developed, optimizing drug efficacy and minimizing off-target effects. Combining multi-omics data for the systemic evaluation of off-target effects and utilizing high-throughput screening methods will be crucial for assessing the safety of m^6^A-targeting drugs.

The complexity of m^6^A’s role across different tumor types complicates its use as a therapeutic target as its effects may vary, either inhibiting or promoting cancer progression depending on the context. The regulation of m^6^A involves multiple enzymes, such as methyltransferases (e.g., METTL3) and demethylases (e.g., FTO, ALKBH5), whose interplay adds another layer of complexity to its functional understanding and therapeutic targeting. Moreover, individual variability in genetic backgrounds, tumor types, and m^6^A expression levels requires personalized therapeutic approaches, as differences in m^6^A modifications between tumor and normal tissues may lead to unintended toxicity. The lack of specific and effective drugs for targeting m^6^A remains a major hurdle, as existing inhibitors often lack selectivity and may interfere with critical biological processes. Additionally, the dynamic nature of m^6^A modifications, influenced by various factors such as the tumor microenvironment and disease progression, makes it challenging to predict therapeutic outcomes. The potential side effects and toxicity associated with modulating m^6^A, such as immune suppression or liver damage, further complicate its clinical application. Finally, while m^6^A’s potential has been demonstrated in laboratory studies, the lack of substantial clinical trial data on its impact in cancer therapy makes its translation into clinical practice difficult. Overall, while targeting m^6^A holds promise, overcoming these challenges will require further research and the development of more specific, efficient, and safe therapeutic strategies.

### 6.4. Causes of Heterogeneity of m^6^A Modification and Future Research Directions

N^6^-methyladenosine (m^6^A) methylation has been shown to play a crucial role in cancer biology, influencing various processes such as gene expression regulation, tumor progression, and metastasis. However, its role in different tumors, and even within the same tumor type, can be contradictory, which complicates the development of m^6^A-targeted cancer therapies. Some key reasons for these contradictions and suggestions for future research directions are as follows: 1. Tumor heterogeneity. Tumors are often heterogeneous, consisting of subpopulations of cells with distinct molecular and genetic profiles. These variations can result in different responses to m^6^A modifications. In some cancer cells, m^6^A may promote tumor growth by enhancing the gene expression of oncogenes, while in other cells, it may suppress tumor progression by regulating tumor suppressors. Therefore, it is important to conduct single-cell RNA sequencing studies to better understand how m^6^A modification affects different subpopulations within a tumor. This will help identify specific tumor cell types that are more responsive to m^6^A modulation, enabling personalized therapies. 2. Differential expression of m^6^A regulators. The expression levels of m^6^A methyltransferases (such as METTL3 and METTL14), demethylases (such as FTO and ALKBH5), and reader proteins (such as YTHDF1 and YTHDF2) vary between different tumors and even between tumor stages. High or low levels of these regulators can lead to opposing effects on m^6^A-mediated RNA modifications, resulting in pro-tumorigenic or anti-tumorigenic outcomes. Therefore, future studies should focus on profiling m^6^A regulators in various tumors to determine how their expression correlates with clinical outcomes. This will aid in identifying key regulators whose manipulation may have therapeutic potential for specific tumor types. Additionally, developing targeted inhibitors or activators of these regulators could provide more precise therapeutic interventions. 3. Microenvironmental influence. The tumor microenvironment (TME), which includes immune cells, fibroblasts, extracellular matrix components, and various signaling molecules, can significantly influence m^6^A modifications. For example, hypoxic conditions in tumors have been shown to alter the expression of m^6^A regulators and contribute to altered gene expression, promoting tumor survival and metastasis. However, the TME can also induce m^6^A-mediated regulation of immune responses, affecting the tumor’s immune evasion strategies. Therefore, to better understand how the TME affects m^6^A dynamics, researchers should examine the interplay between tumor cells and immune cells in the microenvironment. Moreover, studies should investigate the potential of combining m^6^A-targeted therapies with immunotherapy to improve therapeutic outcomes by reprogramming immune responses. 4. The dual role of m^6^A in tumor suppression and promotion. m^6^A modification has been reported to both inhibit and promote cancer progression depending on the context. For instance, m^6^A methylation can regulate the stability of tumor suppressor genes and oncogenes, with different tumor types showing opposing effects. In some cancers, m^6^A enhances the stability of oncogenic mRNAs, promoting cell proliferation, while in others, it may inhibit tumor growth by destabilizing pro-cancerous transcripts. Therefore, in-depth functional studies are needed to explore the specific mechanisms through which m^6^A affects tumor suppression and promotion in different cancer types. Investigating how m^6^A regulators interact with other key molecular pathways, such as those involved in cell cycle regulation and apoptosis, will help identify therapeutic targets that can modulate the dual role of m^6^A in tumor progression. 5. Post-transcriptional modifications and feedback loops. m^6^A affects RNA splicing, translation, and decay, and it may engage in complex feedback loops that influence tumor behavior. For example, the m^6^A-mediated regulation of certain RNA transcripts may lead to changes in the expression of m^6^A regulators themselves, further modulating tumorigenic processes. These feedback mechanisms can result in varying effects in different tumor contexts. Therefore, to uncover the full extent of m^6^A’s impact, it will be important to study the interactions between m^6^A-modified transcripts and their regulators. Comprehensive multi-omics analyses, including transcriptomics, proteomics, and epigenomics, could provide insights into the feedback loops involving m^6^A and their role in tumor biology.

The contradictory effects of m^6^A in different tumor types, as well as its complex regulation and interaction with various tumor-related pathways, pose significant challenges for its therapeutic targeting. To address these issues, future research should focus on understanding the context-dependent roles of m^6^A, identifying key regulators, and exploring combinatory strategies with other therapeutic modalities. This will pave the way for the development of more effective and personalized cancer treatments targeting m^6^A.

## Figures and Tables

**Table 1:** The core m^6^A catalysis enzymes of different nucleic acid types.

m^6^A Enzyme	Nucleic Acid Type	Ref.
N6AMT1 (Me) ALKBH1 (D-me)	DNA	[8]
ZCCHC4 (Me)	28S rRNA	[9]
METTL5	18S rRNA	[10]
METTL16	snRNA	[11]
METTL3/METTL14 (Me)	mRNA	[12]
ALKBH5/FTO (D-me)		[13]

Various nucleic acid types exhibit distinct m^6^A methylation and demethylation enzymes. Specifically, N6AMT1 is involved in the m^6^A methylation of DNA, ALKBH1 is responsible for the m^6^A demethylation of DNA, the enzyme complex containing METTL3/METTL14 as the core is responsible for the m^6^A methylation of mRNA, and ALKBH5 and FTO are responsible for the m^6^A demethylation of mRNA. Additionally, ZCCHC4 is responsible for the m^6^A methylation of 28S rRNA, METTL5 is responsible for the m^6^A methylation of 18S rRNA, and METTL16 is responsible for the m^6^A methylation of snRNA. (N6AMT1: N6 adenine-specific DNA methyltransferase 1; Me: methylation; D-me: demethylation).

**Table 2:** Roles of m^6^A methylation on mRNA in tumor cells.

Effect	Enzyme	Disease	Organ	Genes/Signaling	Ref.
Promote	METTL3 ↑	Osteosarcoma (OS)	Bone	LEF1↑ and the activity of Wnt/β-catenin signaling pathway ↑	[44]
Promote	METTL3 ↑	Colorectal cancer (CRC)	Colorectum	Cyclin E1 ↑	[45]
Promote	METTL3 ↑	Gastric cancer (GC)	Stomach	P300-H3K27 acetylation ↑- METTL3 ↑- HDGF stability ↑ (IGF2BP3)- GLUT4 and ENO2 ↑	[50]
Promote	METTL3 ↑	GC	Stomach	GFI-1 (related to EMT) and α-SMA ↑	[51]
Promote	METTL3 ↑	Prostate cancer (PC)	Prostate	Hedgehog pathway	[72]
Promote	ALKBH5 ↓	PC	Pancreas	WIF-1/ Wnt signaling ↑	[58]
Promote	METTL3 ↑	Bladder cancer (BC)	bladder	AFF4/NF-κB/MYC ↑	[46]
Promote	METTL3 ↑	Osteoarthritis	Joint	NF-kB signaling and ECM synthesis	[116]
Promote	METTL3 ↑	Hepatoblastoma (HB)	Liver	Wnt/β-catenin pathway ↑	[47]
Promote	METTL3 ↑ YTHDF2	Hepatocellular carcinoma (HCC)	Liver	SOCS2/JAK/STAT pathway ↑	[48]
Promote	KIAA1429↑			GATA3 ↓	[49]
Promote	YTHDF1 ↑	Merkel cell carcinoma (MCC)	Skin	---	[29]
Inhibit	METTL14	Renal cancer (RCC)	Kidney	P2RX6/Ca^2+^/p-ERK1/2/MMP9 signaling ↓	[52]
Inhibit	METTL3/14 FTO	Glioblastoma	Brain	Cell proliferation, differentiation, and DNA damage response	[74]
Inhibit	ALKBH5/FTO ↑ METTL3/14 ↓ YTHDF1-3 ↓	GC	Stomach	Wnt and PI3K-Akt signaling ↑	[53]
Inhibit	FTO ↑	Acute myeloid leukemia (AML)	Bone	P53/apoptotic pathway ↓	[54]
Inhibit	FTO ↑	Cervical cancer	Cervix	E2F1 and MYC	[55]
Inhibit	YTHDF1	Ocular melanoma	Uveal or conjunctival	HINT2 ↑	[117]

The roles of m6A RNA-related enzymes in influencing tumor progression by modifying mRNA expression are summarized in the table. In order to eliminate possible differences in action at different sites, we further reflected the corresponding action sites and mechanisms of m^6^A RNA-modification-related enzymes. α-SMA: α-smooth muscle actin; EMT: epithelial–mesenchymal transition. ↑ increase, ↓ decrease.

**Table 3:** Clinical trials for tumor therapy targeting m^6^A.

Registration Number	Drug	Target	Indications	Stage	Country
NCT04989335	CS1, fludarabine, clofarabine [126]	FTO	Relapsed or refractory AML	II	Israel
NCT05584111	STC-15 [133]	METTL3	AML, advanced solid tumor, hematological neoplasm	I	United States
NCT03384914	pUMVC3-IGFBP2-HER2-IGF1R	IGFBP2	Breast cancer	II	United States
NCT05794659	AST-201	IGFBP2	Peritoneal neoplasm, ovarian neoplasm, fallopian tube cancer, metastatic ovarian cancer	II	United States
	METTL3 inhibitor	METTL3	AML	Preclinical	United States
	EP-102	METTL3	Non-small-cell lung cancer, acute myeloid leukemia, squamous cell carcinoma, ovarian tumor	Preclinical	Belgium
	Next-generation CS1	FTO	Malignant neoplasm	Preclinical	Australia

Data from the entropy medicine database: https://pharma.bcpmdata.com/ (accessed on 1 February 2025) https://10.1080/14740338.2024.2390640.

**Table 4:** Drugs not yet in clinical trials for tumor therapy targeting m^6^A.

Drug	Target	Molecular Formula	Indications	Country
MV-1035	ALKBH5	C14 H14 N2 O S	Glioblastoma	United States
RSM-3	METTL3	C124 H214 N54 O26 S3	Malignant tumor	China
UZH-2	METTL3	C27 H37 F2 N7 O	Malignant tumor	Switzerland
UZH-1a	METTL3	C32 H42 N6 O3	AML	United States
STM-3480	METTL3	C24 H25 N5 O2	Malignant tumor	Britain
D272-0843	METTL3	C21 H17 Cl F N3 O2	Malignant tumor	China
EP-201	IGFBP2	--	Metastatic ovarian cancer	United States

Data from the entropy medicine database: https://pharma.bcpmdata.com/ (accessed on 1 February 2025) https://10.1080/14740338.2024.2390640.

## Abbreviations

Abbreviation	Full Form/Description	Abbreviation	Full Form/Description
ALKBH5	AlkB homolog 5	NSCLC	Non-small-cell lung cancer
α-SMA	α-smooth muscle actin	OS	Osteosarcoma
AML	Acute myeloid leukemia	PC	Prostate cancer
BC	Bladder cancer	P2RX6	Purinergic receptor P2X, ligand-gated ion channel 6
BRCA	Breast cancer gene	PI3K-Akt	Phosphoinositide 3-kinase-Akt
CBX8	Chromobox 8	PDAC	Pancreatic ductal adenocarcinoma
CCNE1	Cyclin E1	PRAD	Prostate adenocarcinoma
CDK2	Cyclin-dependent kinase 2	PRC1	Polycomb repressive complex 1
COAD	Colon adenocarcinoma	RBM15	RNA-binding motif protein 15
CRC	Colorectal cancer	READ	Rectum adenocarcinoma
DCs	Dendritic cells	RCC	Renal cancer
EMT	Epithelial–mesenchymal transition	SRF	Serum response factor
ESCs	Embryonic stem cells	STAD	Stomach adenocarcinoma
FMRP	Fragile X mental retardation protein	TLR	Toll-like receptor
FTO	Fat mass and obesity-associated protein	Tregs	Regulatory T cells
GATA3	GATA-binding protein 3	USP7	Ubiquitin-specific peptidase 7
GC	Gastric cancer	WIF-1	Wnt inhibitory factor 1
GSCs	Glioblastoma stem cells	WTAP	Wilms tumor 1-associating protein
HB	Hepatoblastoma	Wnt	Wingless-related integration site
HCC	Hepatocellular carcinoma	YAP	Yes-associated protein
HDGF	Hepatoma-derived growth factor	YAP1	Yes-associated protein 1
HSPCs	Hematopoietic stem and progenitor cells	YTHDC1	YTH domain containing 1
HIF-1	Hypoxia-inducible factor 1	YTHDC2	YTH domain containing 2
HIF-1α	Hypoxia-inducible factor 1 alpha	YTHDF1	YTH N6-methyladenosine RNA binding protein 1
ICB	Immune checkpoint blockade	YTHDF2	YTH N6-methyladenosine RNA binding protein 2
IGF2BP1	Insulin-like growth factor 2 mRNA binding protein 1	YTHDF3	YTH N6-methyladenosine RNA binding protein 3
IGF2BP2	Insulin-like growth factor 2 mRNA binding protein 2	ZC3H13	Zinc finger CCCH-type containing 13
IGF2BP3	Insulin-like growth factor 2 mRNA binding protein 3	ccRCC	Clear-cell renal cell carcinoma
JAK/STAT	Janus kinase/signal transducer and activator of transcription	circRNA	Circular RNA
KIAA1429	KIAA1429 gene	eIF3	Eukaryotic translation initiation factor 3
LGR5	Leucine-rich repeat-containing G-protein coupled receptor 5	m6A	N6-methyladenosine
LIHC	Liver hepatocellular carcinoma	mRNA	Messenger RNA
LUAD	Lung adenocarcinoma	mTOR	Mechanistic target of rapamycin
LUSC	Lung squamous cell carcinoma	miRNA	MicroRNA
LncRNA	Long non-coding RNA		
LncXIST	Long non-coding RNA X-inactive specific transcript		
MDSCs	Myeloid-derived suppressor cells		
METTL14	Methyltransferase-like protein 14		
METTL3	Methyltransferase-like protein 3		
MYC	Myelocytomatosis oncogene		
NF-κB	Nuclear factor kappa-light-chain-enhancer of activated B cells		
